# Supplementary material for: Epithelial coxsackievirus adenovirus receptor promotes house dust mite-induced lung inflammation
Source: Nat Commun. 2022 Oct 27;13:6407. doi: 10.1038/s41467-022-33882-w (PMC9613683; doi:10.1038/s41467-022-33882-w)

Supplementary Figure 1

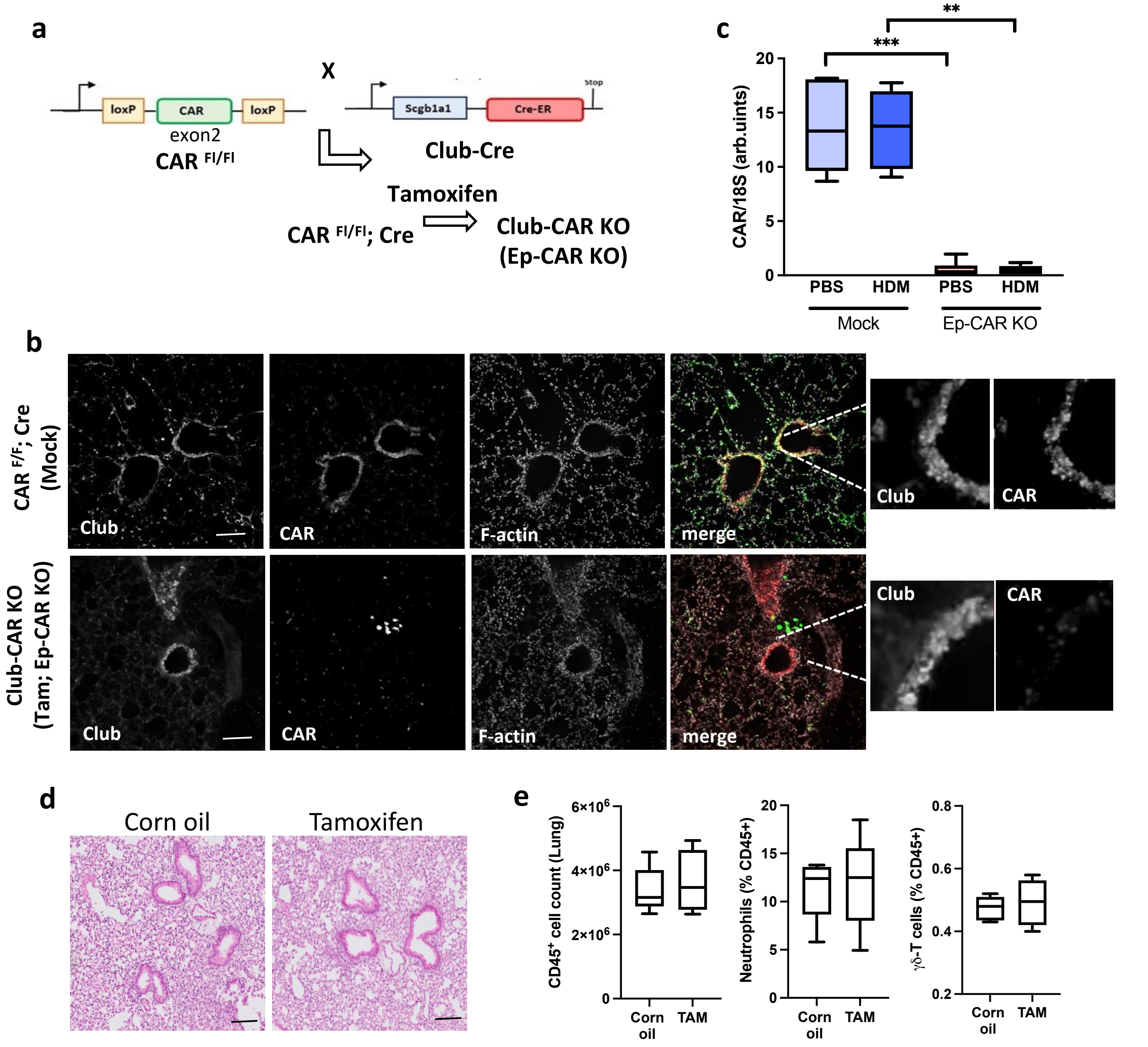

Supplementary Figure 1: Generation of a lung epithelial-specific CAR knockout mouse

(A). Schematic illustrating breeding scheme and Tamoxifen treatment to generate a lung epithelial KO mouse. $CAR^{fl/fl}$ mice were crossed with a Scgb1a1-CreERT to enable recombination and CAR depletion specifically in bronchiolar Club following intra-peritoneal administration of Tamoxifen (Ep-CAR KO) **(B)** Representative confocal images of lungs from a $CAR^{fl/fl}$ mouse crossed with a Scgb1a1-CreERT in the absence (Mock) or presence of Tamoxifen (Ep-CAR KO) stained for a club cell marker, CAR and F-actin. Scale bars: 100 μm. **(C)** CAR levels quantified by qPCR from total lungs. Levels of 18S were used to normalised values. Graph of data from lungs of 5 mice from each group; representative of 3 independent experiments. **(D)** Representative images of H&E sections from lungs of C57Bl/6 mice treated with corn oil or Tamoxifen for 2 weeks. Scale bars: 100 μm. **(E)** Total $CD45^+$ cells, % of neutrophils and $\gamma\delta$Tcells in digested lung tissue from C57Bl/6 mice treated with corn oil or Tamoxifen for 1 week, harvested 3 weeks after initial dosing; 6 mice per group. All values in graphs show median (line) with 25/75 percentiles (box) and min/max values. two-way ANOVA with Tukey's post hoc test was used to test significance in (e). P values: **=p<0.01, ***= p<0.0005.

Supplementary Figure 2

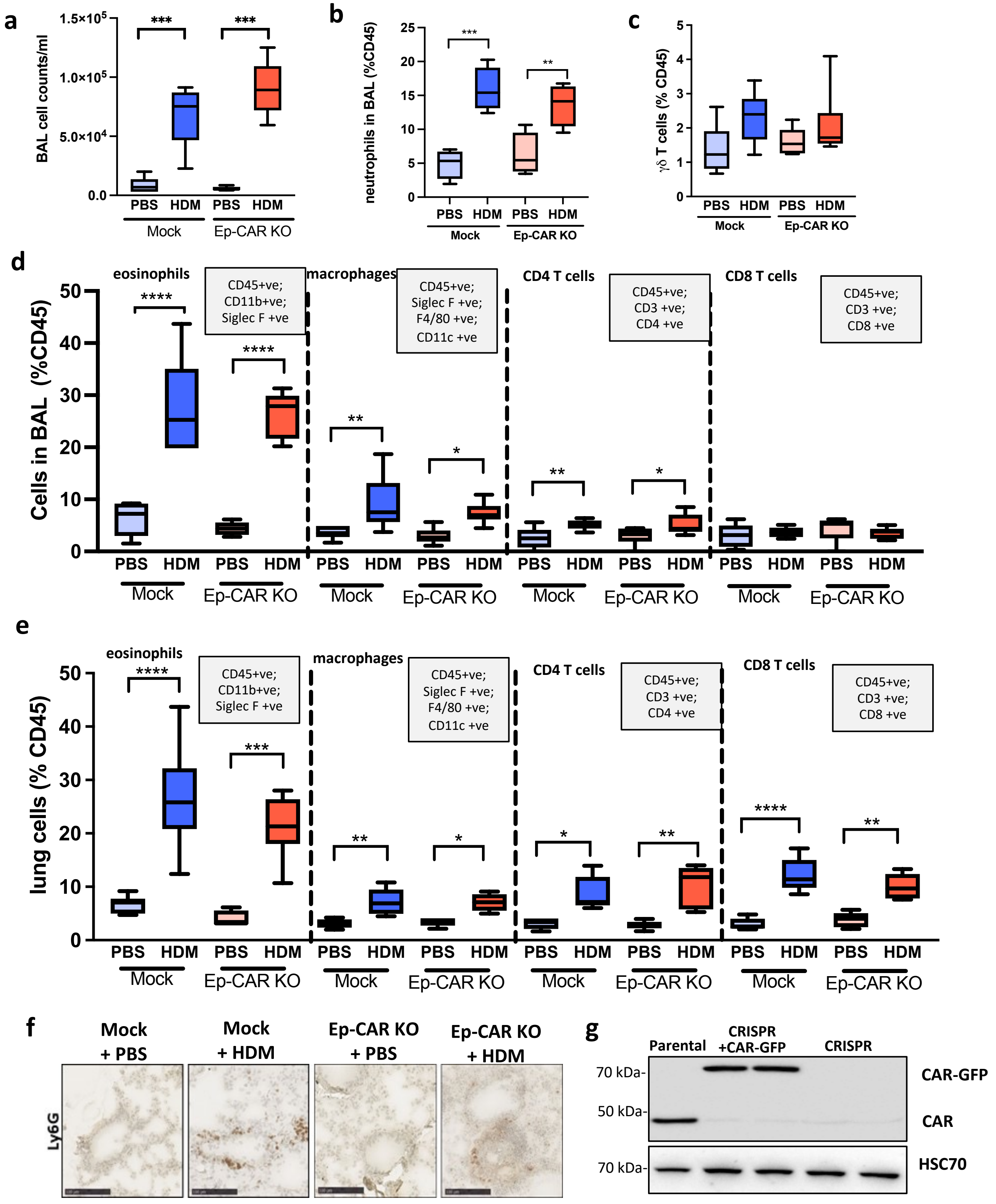

Supplementary Figure 2: Immune profiling lungs in CAR knockout mice

(A) Total cell counts in BAL fluid and **(B)** % of neutrophils and **(C)** γδ T cells within BAL. **(D,E)** % of eosinophils, macrophages, CD4+ and CD8+ T cells in (D) BAL and (E) digested lung tissue from mice from the four different experimental groups used. Graphs show pooled data from 3 independent experiments with 5 mice per group per experiment. **(F)** Representative images of Ly6G staining (brown) of lung sections from indicated conditions. Scale bar: 100 μm. **(G)** Western blot showing endogenous CAR levels in parental 16HBE cells, two CAR CRISPR cell cultures and two CRISPR cultures rescued with GFP-CAR lentivirus. All values in graphs show median (line) with 25/75 percentiles (box) and min/max values. Unpaired 2-tail student's T-tests were performed. P values * =p<0.05,**=p<0.01, ***= p<0.0005,****= p<0.0001.

Supplementary Figure 3

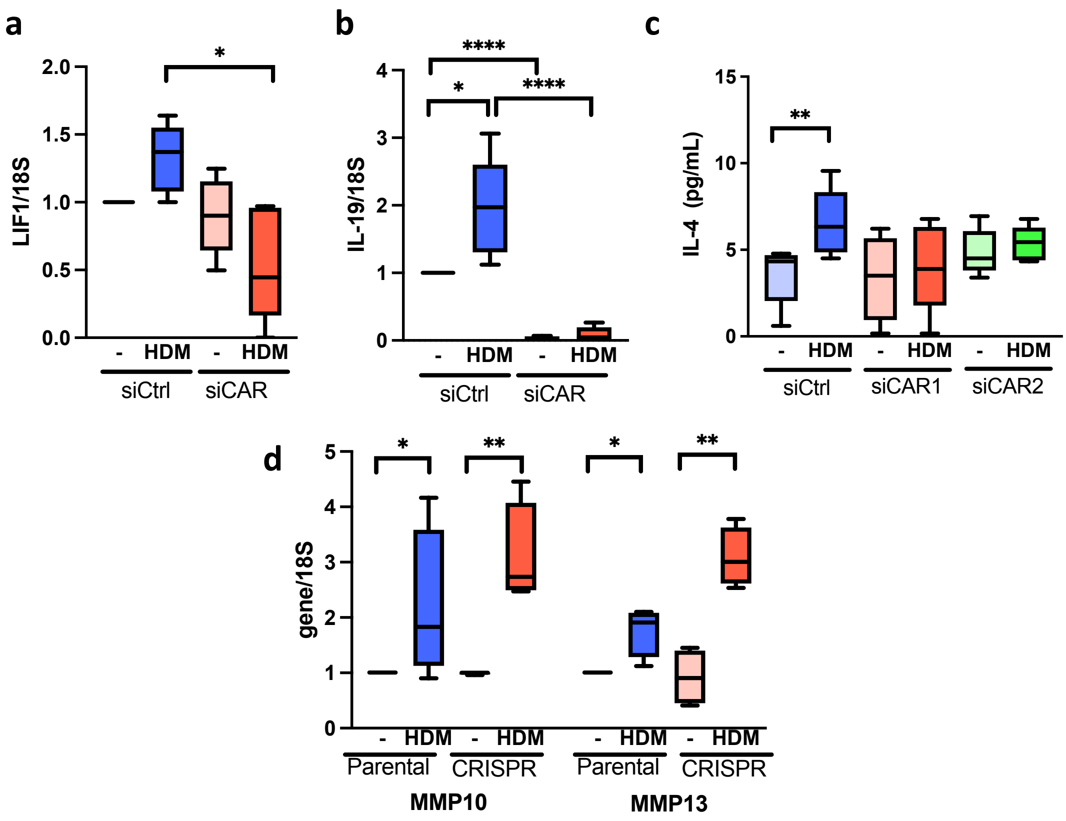

Supplementary Figure 3: Validation of cytokine array data

(A,B) LIF1 (**A**) and IL19 (**B**) transcript levels measured by qPCR in 16HBE cells treated with control or CAR siRNA +/- HDM for 24h. 18S was used to normalise values. Graph shows pooled date from 5 independent experiments. **(C)** Analysis of secreted IL-4 levels from supernatants of control or CAR siRNA treated 16HBE cells +/- HDM for 24h as measured by ELISA. Graph shows date from 3 replicates from one experiments; 3 independent experiments were performed. **(D)** MMP10 and MMP13 transcript levels in parental 16HBE and CAR CRISPR cells +/- HDM for 24h measured by qPCR. 18S was used to normalise values. Graph shows pooled date from 4 independent experiments. All values in graphs show median (line) with 25/75 percentiles (box) and min/max values. One-way ANOVA with Dunnett's post hoc test was used to test significance in (a-c); . Unpaired 2-tail student's T-tests were performed within datasets for (d). P values * =p<0.05, **=p<0.01, ****= p<0.0001.

Supplementary Figure 4

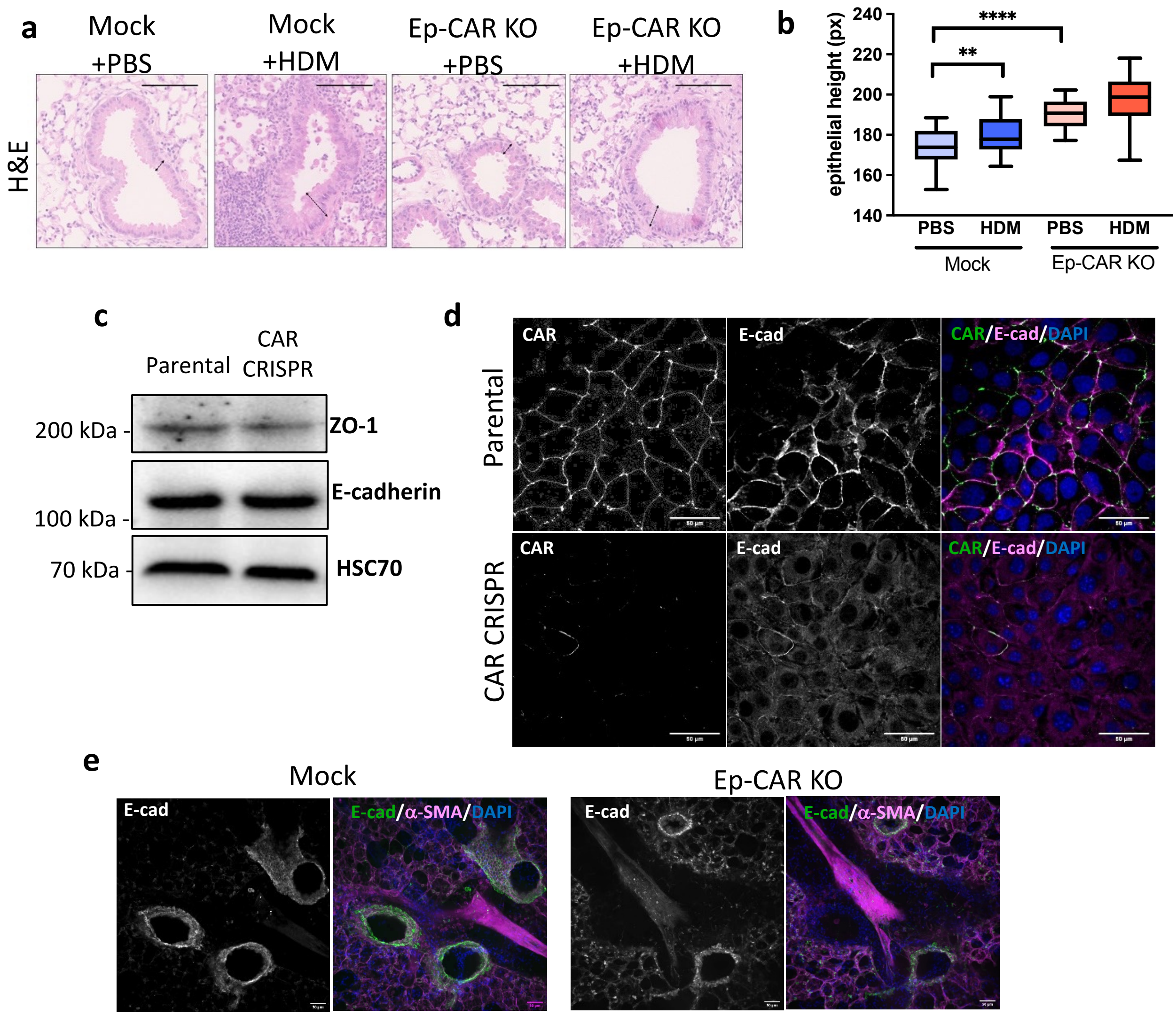

Supplementary Figure 4: CAR contributes to lung epithelial cell morphology and integrity
(A) Representative H&E images of lungs from Mock and Ep-CAR KO mice treated with PBS or HDM over 5 weeks. Arrows show examples of measured epithelial cell heights. Scale bars: 100 μm. (**B)** Quantification of epithelial height from images as in (A). Data shown is from 5 airways, from 3 independent experiments with 5 mice per group. (**C)** Western blot of lysates from 16HBE parental and CAR CRISPR cells probed for ZO-1 and E-cadherin. (**D)** Representative confocal images of 16HBE parental and CAR CRISPR cells stained for E-cadherin (magenta), CAR (green) and DAPI (blue). Scale bars: 50 μm. (**E)** Representative confocal Z-projections of PLCS from Mock and Ep-CAR KO mice treated with PBS, fixed and stained for E-cadherin (green), αSMA (magenta) and DAPI (blue). Scale bars: 10 μm. Values in graph show median (line) with 25/75 percentiles (box) and min/max values. One-way ANOVA with Dunnett's post hoc test was used to test significance . P values,**=p<0.01,****= p<0.0001.

Supplementary Figure 5

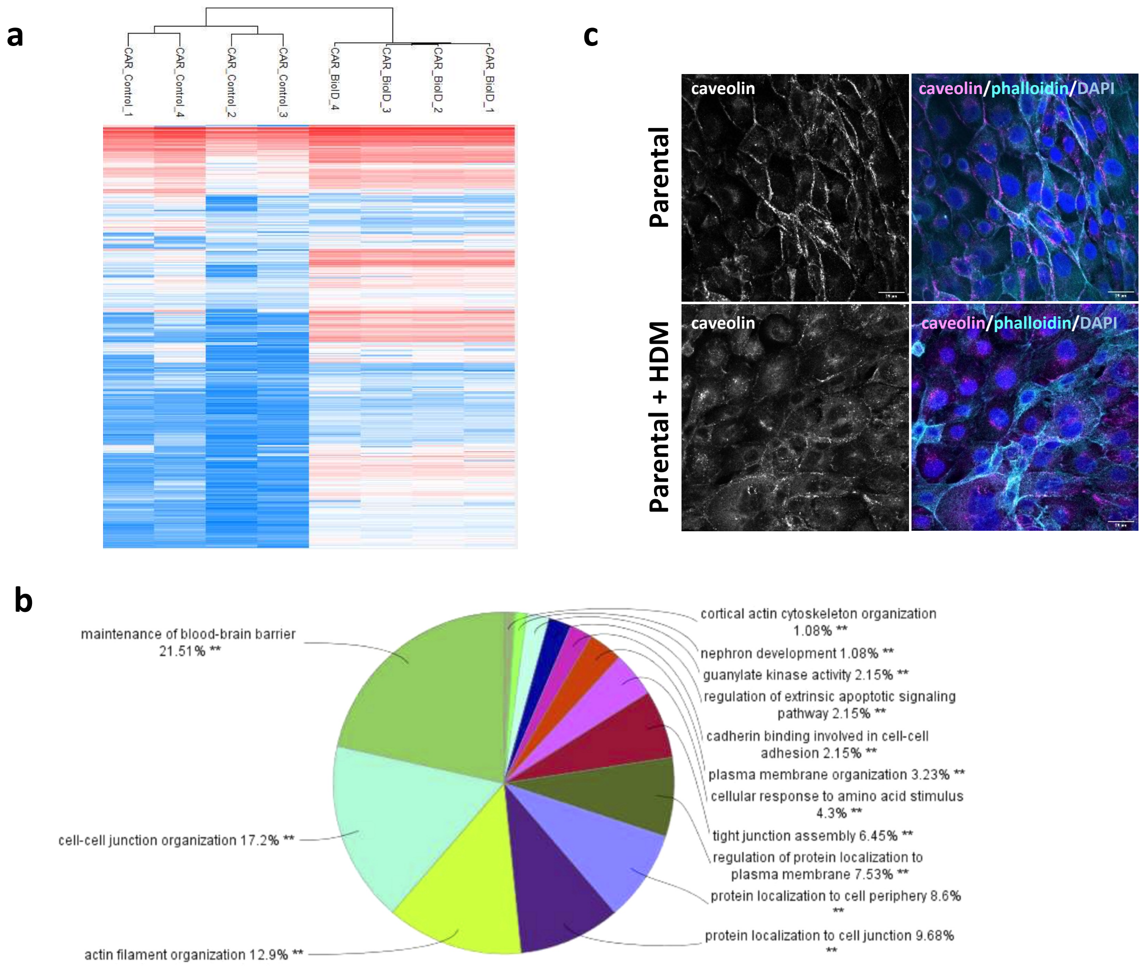

Supplementary Figure 5: Novel CAR binding partners revealed by BioID
(A) Overview of IBAQ intensity data from BioID analysis of 16HBE CAR CRISPR cells expressing CAR-BirA. (**B**) ClueGO analysis of top 100 enriched proteins from CAR BioID analysis. The area of the wedges is proportional to the percentages of GO terms; functions reported in the pie chart are those with the highest percentages of functional categories. (**C**) Representative confocal images of 16HBE parental cells +/-HDM for 24h, fixed and stained for caveolin-1 (magenta), F-actin (cyan) and DAPI (blue). Scale bars: 50 μm.

Supplementary Figure 6

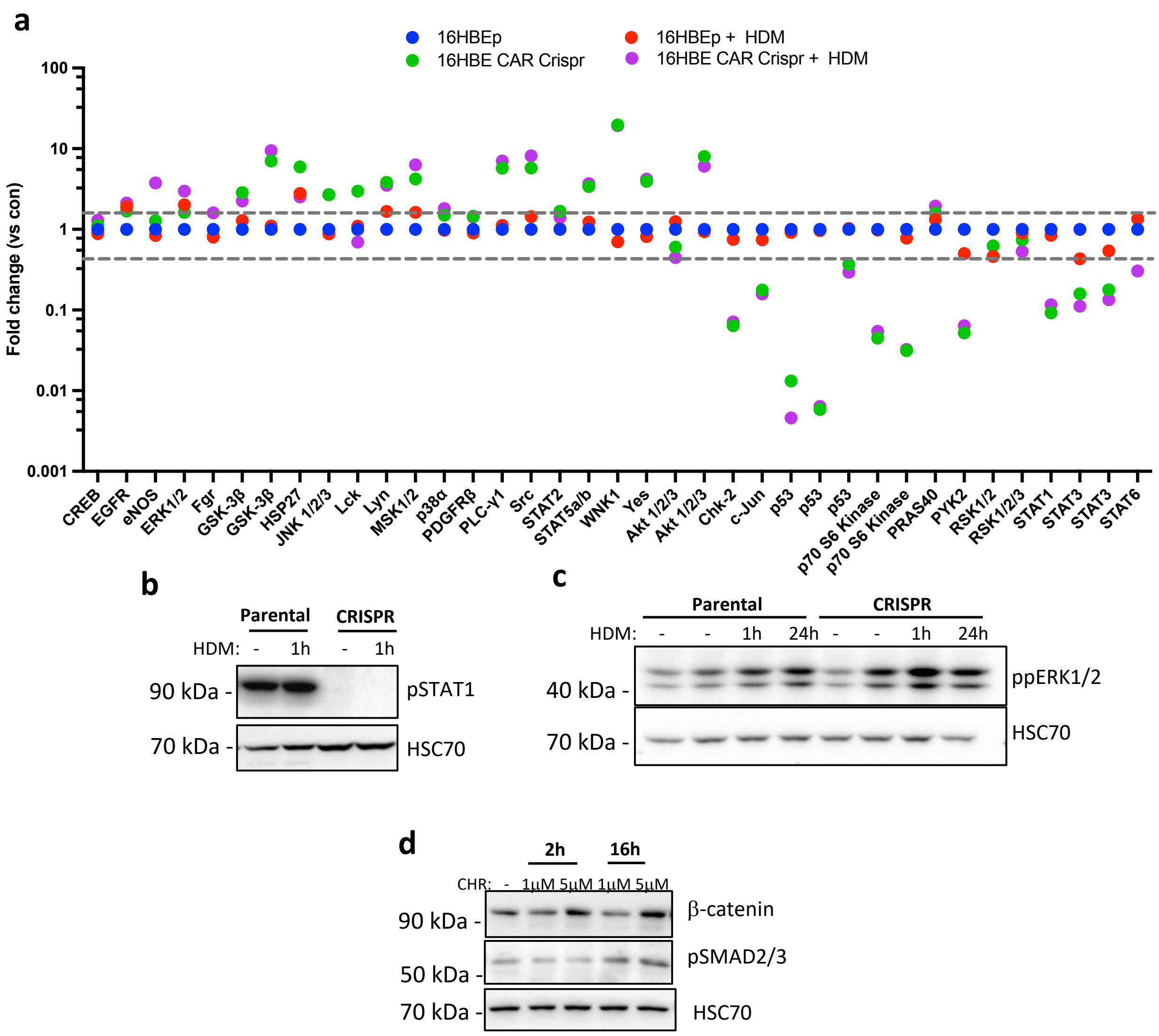

Supplementary Figure 6: CAR-dependent intracellular signalling pathways in the lung epithelium
(A) Results from the human phospho-kinase array in parental and CAR CRISPR 16HBE cells. Means of 3 replicates per sample are shown. (**B)** Representative western blot of pSTAT1 in lysates from cells as in (A). (**C)** Representative western blot of ppERK1/2 in cells as in (A) treated with HDM for 1h or 24h. (**D)** Representative western blot of β-catenin and pSMAD2/3 in parental 16HBE cells treated with GSK3β inhibitor CHIR-99021 at 1μm or 5μm for 2h or 16h. All blots shown are representative of at least 3 independent experiments.

Supplementary Figure 7

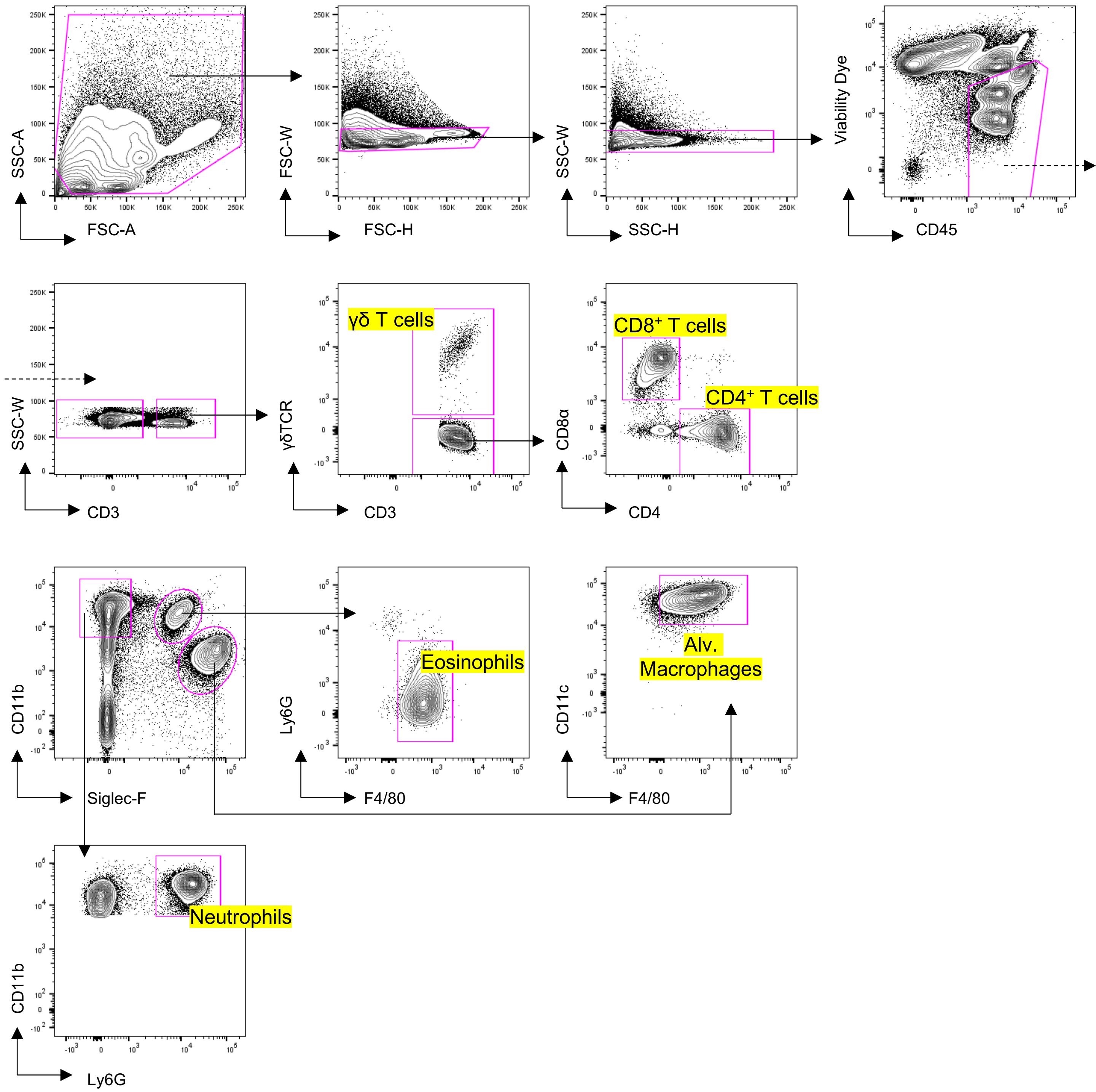

Supplementary Fig 7: FACS sequential gating/sorting strategy.
Representative contour flow cytometry plots demonstrating the gating strategy employed to identify immune cell content in BAL and lung homogenates. Single, live, CD45^{+} leucocytes were identified by cellular size/scatter and viability dye exclusion as shown. Live leukocyte populations of interest were identified based on differential expressions of the indicated markers: **γδ T cells** = CD3^{+} γδ TCR^{+}, **CD4^{+} αβ T cells** = CD3^{+} γδ TCR^{-}CD8α^{-}CD4^{+} , **CD8^{+} αβ T cells** = CD3^{+} γδ TCR^{-}CD8α^{+}CD4^{-} , **Neutrophils** = CD3^{-} CD11b^{hi}Siglec-F^{-}Ly6G^{+}, **Eosinophils**= CD3^{-}CD11b^{hi}Siglec-F^{+}Ly6G$^{low/-}$F4/80$^{Low/+}$ **Alveolar (Alv) Macrophages** = CD3^{-} CD11b^{low}Siglec-F^{+} CD11c^{Hi}F4/80$^{+/Hi}$

Supplementary data source data – full western blots

Fig S2F CAR

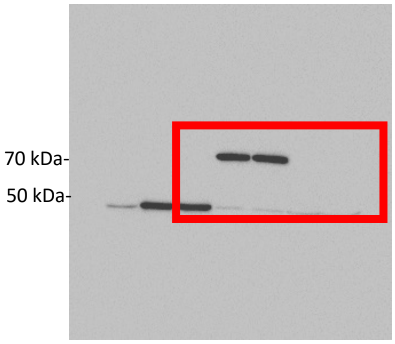

Fig S2F HSC70

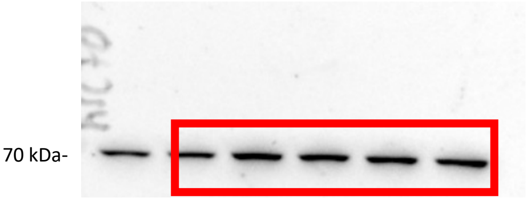

Fig S4C HSC70

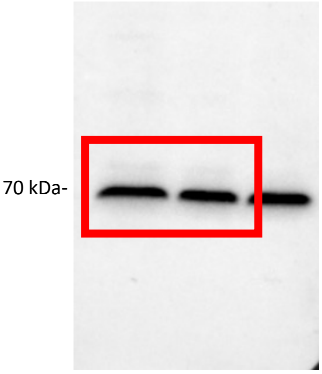

Fig S4C ZO-1

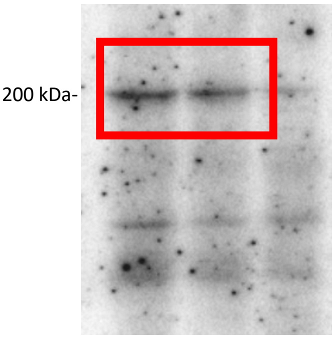

Fig S4C Ecad

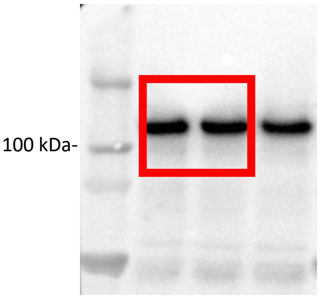

Fig S6B pSTAT1

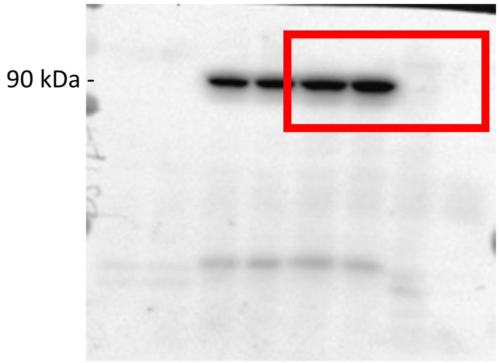

Fig S6B HSC70

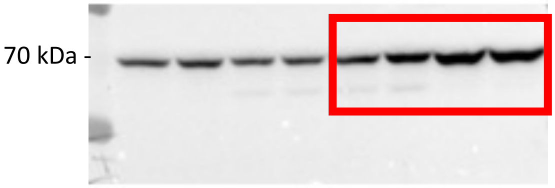

Fig S6C ppERK1/2

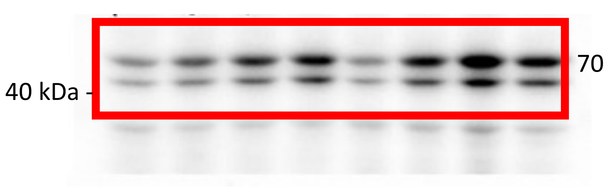

Fig S6C HSC70

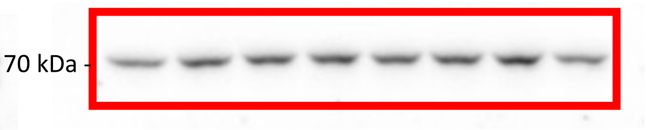

Fig S6D β-catenin

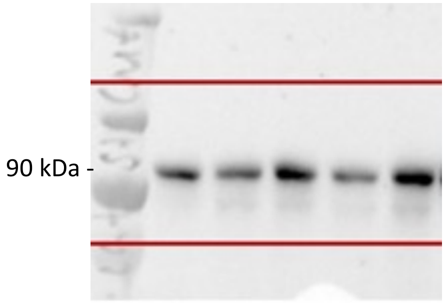

Fig S6D pSMAD2/3

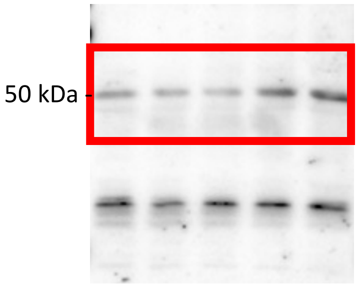

Fig S6D HSC70

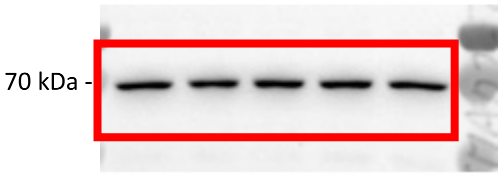

Supplement: Supplementary file 1 — Supplementary Information [file 41467_2022_33882_MOESM1_ESM.pdf]
