# Supplementary material for: Epithelial coxsackievirus adenovirus receptor promotes house dust mite-induced lung inflammation
Source: Nat Commun. 2022 Oct 27;13:6407. doi: 10.1038/s41467-022-33882-w (PMC9613683; doi:10.1038/s41467-022-33882-w)
Supplement: Supplementary file 3 — Description of Additional Supplementary Files [file 41467_2022_33882_MOESM3_ESM.pdf]

Supplementary Data 1: Proteins identified in complex with CAR by BioID
Excel spreadsheet detailing the results of BioID analysis of CAR-BirA expressing 16HBE cells. Full dataset is provided (tab: BioID enriched proteins') and the Top 100 (tab: 'Top 100") ranked by iBAQ CAR_BioID_Control (log2 Ratio) values.
