# Supplementary material for: Epithelial coxsackievirus adenovirus receptor promotes house dust mite-induced lung inflammation
Source: Nat Commun. 2022 Oct 27;13:6407. doi: 10.1038/s41467-022-33882-w (PMC9613683; doi:10.1038/s41467-022-33882-w)
Supplement: Supplementary file 5 — Reporting Summary [file 41467_2022_33882_MOESM5_ESM.pdf]

nature portfolio

Corresponding author(s): Maddy Parsons

Last updated by author(s): Jan 14, 2022

Reporting Summary

Nature Portfolio wishes to improve the reproducibility of the work that we publish. This form provides structure for consistency and transparency in reporting. For further information on Nature Portfolio policies, see our Editorial Policies and the Editorial Policy Checklist.

Statistics

For all statistical analyses, confirm that the following items are present in the figure legend, table legend, main text, or Methods section.

n/a	Confirmed	
☐	☒	The exact sample size (n) for each experimental group/condition, given as a discrete number and unit of measurement
☐	☒	A statement on whether measurements were taken from distinct samples or whether the same sample was measured repeatedly
☐	☒	The statistical test(s) used AND whether they are one- or two-sided<br>*Only common tests should be described solely by name; describe more complex techniques in the Methods section.*
☒	☐	A description of all covariates tested
☐	☒	A description of any assumptions or corrections, such as tests of normality and adjustment for multiple comparisons
☐	☒	A full description of the statistical parameters including central tendency (e.g. means) or other basic estimates (e.g. regression coefficient) AND variation (e.g. standard deviation) or associated estimates of uncertainty (e.g. confidence intervals)
☐	☒	For null hypothesis testing, the test statistic (e.g. F, t, r) with confidence intervals, effect sizes, degrees of freedom and P value noted<br>*Give P values as exact values whenever suitable.*
☒	☐	For Bayesian analysis, information on the choice of priors and Markov chain Monte Carlo settings
☒	☐	For hierarchical and complex designs, identification of the appropriate level for tests and full reporting of outcomes
☒	☐	Estimates of effect sizes (e.g. Cohen's d, Pearson's r), indicating how they were calculated

Our web collection on statistics for biologists contains articles on many of the points above.

Software and code

Policy information about availability of computer code

Data collection	Microscopy data was collected using NIS Elements AR(4.5). FACS data was collected using Attune NxT flow cytometer (ThermoFisher Scientific)
Data analysis	Microscopy data was analysed using NIS Elements AR (v4.5) and FIJI (v1.53). Raw proteomics data was analysed using MaxQuant software package (v1.6.3.4).Western blots were analysed and processed using Image Lab (v5.2.1, Bio-Rad Laboratories). FACS data was analysed using FlowJo software (v9.0). Statistical analysis was performed in Prism v8, v9 (GraphPAD Software), EVOS2 software was used for data collection (v 2.0.2094.0)

For manuscripts utilizing custom algorithms or software that are central to the research but not yet described in published literature, software must be made available to editors and reviewers. We strongly encourage code deposition in a community repository (e.g. GitHub). See the Nature Portfolio guidelines for submitting code & software for further information.

Data

Policy information about availability of data

All manuscripts must include a data availability statement. This statement should provide the following information, where applicable:

- Accession codes, unique identifiers, or web links for publicly available datasets
- A description of any restrictions on data availability
- For clinical datasets or third party data, please ensure that the statement adheres to our policy

Complete BioID analysis datasets are provided in Supplementary Data 1.
The BioID mass spectrometry proteomics data have been deposited to the ProteomeXchange Consortium via the PRIDE partner repository with the dataset identifier PXD029237

Source data are provided with this paper.

Field-specific reporting

Please select the one below that is the best fit for your research. If you are not sure, read the appropriate sections before making your selection.

[x] Life sciences   [ ] Behavioural & social sciences   [ ] Ecological, evolutionary & environmental sciences

For a reference copy of the document with all sections, see nature.com/documents/nr-reporting-summary-flat.pdf

Life sciences study design

All studies must disclose on these points even when the disclosure is negative.

Sample size	Experimental sample sizes were determined based on extensive prior experience of identical or similar cell biology or biochemical assays or prior experiments using the HDM challenge model in vivo (see for example DOI: 10.1183/09031936.00022908).
Data exclusions	No data was excluded from analysis
Replication	All experiments were repeated on at least 3 independent occasions as detailed in figure legends. Only data that was reproducible across all experiments was presented.
Randomization	samples were randomly allocated to experimental groups.
Blinding	investigators were blinded during data acquisition and/or analysis as indicated in the manuscript.

Reporting for specific materials, systems and methods

We require information from authors about some types of materials, experimental systems and methods used in many studies. Here, indicate whether each material, system or method listed is relevant to your study. If you are not sure if a list item applies to your research, read the appropriate section before selecting a response.

Materials & experimental systems

n/a	Involved in the study	
[ ]	[x]	Antibodies
[ ]	[x]	Eukaryotic cell lines
[x]	[ ]	Palaeontology and archaeology
[ ]	[x]	Animals and other organisms
[x]	[ ]	Human research participants
[x]	[ ]	Clinical data
[x]	[ ]	Dual use research of concern

Methods

n/a	Involved in the study	
[x]	[ ]	ChIP-seq
[ ]	[x]	Flow cytometry
[x]	[ ]	MRI-based neuroimaging

Antibodies

Antibodies used

p-CAR Thr290/Ser293 polyclonal antibody was previously described and was developed by Perbioscience (Thermofisher) using the peptide Ac- RTS (pT)AR(pS)YIGSNH-C and was affinity purified before use.

Santa Cruz Technology - Anti-CAR (H300, discontinued), Anti HSC70 (sc-7298, clone B6).

Cell Signaling Technology - Phospho-p44/42 MAPK (Thr202/Tyr204 (9101); p44/42 MAPK (4695; clone 137F5); Phospho-PKCdelta Tyr311 Antibody (2055). Phospho-Stat1 Tyr701 (9167, clone 58D6); rabbit anti-E-Cadherin (24E10 – 3195)

Abcam - alpha smooth muscle Actin (1A4); Collagen I (clone EPR7785, ab138492); caveolin1 (clone E249, ab32577); Ly6g (clone EPR22909-135; ab238132); IL19 (clone EPNCIR168, ab154187); E-Cadherin antibody (clone DECMA-1; ab11512)

Sigma - Anti-fibronectin (F7387, Clone FN-15); Clara/club cell (07-623); ZO-1 (clone 5G6.1, MABT339)

Roche - GFP (11814460001)

R&D Systems - phospho-Ser9 GSK3 (clone 609739, MAB25062), GSK3 (clone 272536, MAB2506); phospho SMAD2/3 (clone 1074A, MAB8935)

DAKO - Anti-mouse HRP (K4000) and anti-rabbit-HRP (K4002)

Invitrogen - Anti-mouse-488 (A11001), Anti-rabbit-488 (A11008), Anti-mouse 568 (A11004), Anti-rabbit 568 (A11011)

Biolegend - TCR- Brilliant violet 421 (clone B1, 331218); CD45-PerCP Cy5 (clone 2D, 368504); CD3-FITC (clone 17A2, 100210); CD4-PE (clone GK1.5, 100408); B220 PECy7 (clone RA3-6B2, 103222); CD8 Alexa647 (clone SK1, 344726); NK1.1 APC-Cy7 (clone PK136, 108724); Ly-6G Brilliant violet 421 (clone 1A8, 127628); CD11c FITC (clone N418, 117306), CD11b PE (clone M1/70 101208)

BD Biosciences - Siglec F-Alexa700 (clone E50-2440, AB_2739097)

Validation

The non-commercial anti pCAR antibody was validated in a previous publications from our laboratory (Morton et al, 2013, 2016) and was routinely tested against CAR negative cell lines or those treated with calyculinA as a positive control. The commercial antibodies were validated by the relevant vendors as detailed on the relevant websites.

Eukaryotic cell lines

Policy information about cell lines

Cell line source(s)

16HBE human bronchial epithelial cells were a gift from Prof D. Gruenert (University of Vermont, US; commercially available from Sigma (SCC150)
HEK 293T cells (used for lentiviral production) were purchased from ATTC (CRL-3216)
Primary human lung fibroblasts were a gift from Prof Jenkins (National Heart and Lung Institute, Imperial College London, UK)
Airway smooth muscle cells were a gift from Dr. Woczcek (King's College London, UK)
HL60 cells were purchased from ATCC (CCL-240)

Authentication

none of the cell lines used were authenticated

Mycoplasma contamination

All cells were tested monthly for mycoplasma and were found to be negative

Commonly misidentified lines
(See ICLAC register)

No commonly misidentified cells were used in this study

Animals and other organisms

Policy information about studies involving animals; ARRIVE guidelines recommended for reporting animal research

Laboratory animals

Scgb1a1-CreERTM were purchased from The Jackson Laboratory. These mice express a tamoxifen-inducible form of cre recombinase from the Scgb1a1 locus (secretoglobin), inducing cre recombinase activity in bronchiolar non-ciliated Club cells. These mice were crossed with mice carrying floxed alleles for CAR (B6;129S2-Cxadrtm1.1Ics) (17). Male and female mice were used for breeding. Only female mice were used for experimentation. Female C57BL/6 mice were purchased from Jackson Labs (strain 027). Intraperitoneal administration of Tamoxifen (Sigma) was used to induce the lung epithelial expression of Cre and excision of CAR. Tamoxifen was administered using 75 mg tamoxifen/kg body mouse weight for 6 consecutive days and lungs were extracted after 2 weeks from the end of the Tamoxifen treatment. Tamoxifen was diluted in corn oil (Sigma). Control mice (mock, no KO generated) received corn oil intraperitoneally. All mice were used between 6 and 12 weeks of age. Mice were housed under 12-hour light/12- hour dark cycles with the lights slowly rising and dimming over a 30min period. Housing temps range between 20 and 23 degC, humidity 45 to 55.

Wild animals

no wild animals were used in this study

Field-collected samples

no field-collected animals were used in this study

Ethics oversight

The use of animals for this study was approved by the Ethical Review Committee at King's College London and the Home Office, UK. All animals were housed in the Biological Support Unit (BSU) located in New Hunt's House at King's College London. All experiments were carried out under project license no. P9672569A and personal license no I0F9CA46A.

Note that full information on the approval of the study protocol must also be provided in the manuscript.

Flow Cytometry

Plots

Confirm that:

- [x] The axis labels state the marker and fluorochrome used (e.g. CD4-FITC).
- [x] The axis scales are clearly visible. Include numbers along axes only for bottom left plot of group (a 'group' is an analysis of identical markers).
- [x] All plots are contour plots with outliers or pseudocolor plots.
- [x] A numerical value for number of cells or percentage (with statistics) is provided.

Methodology

Sample preparation

Bronchoalveolar lavage (BAL) was performed by washing the airways three times with 400 µl of PBS, after centrifugation

	supernatants were stored at –80°C for further analysis and cells resuspended in 500 µl of complete media (RPMI, 10% FCS, 2 mM L-glutamine, 100 U/ml penicillin/streptomycin) (GIBCO, Life Technologies). The left and inferior lung lobes were chopped and digested in complete media supplemented with 0.15 mg/ml collagenase (Type D; Roche) and 25 µg/ml DNase (Type 1; Roche) for 1 hr at 37°C. Tissue was then passed through a 70 µm sieve (BD Bioscience), washed, and resuspended in 1 ml of complete media. in 1 ml of complete media. Red blood cells in 200 µl whole blood were lysed and the remaining leukocytes were washed twice and then resuspended in 1 ml of complete media. Cells were washed and incubated for 20 min with rabbit serum (Sigma-Aldrich) prior to staining for extracellular antigens in 5% FCS / 1% BSA in PBS for 30 min at 4°C. Cells were washed, fixed, and permeabilized using Fix/Perm kit (eBioscience) before being stained for intracellular antigens.
Instrument	Attune NxT flow cytometer (ThermoFisher Scientific)
Software	FlowJo software (Tree Star, Ashland, Ore)
Cell population abundance	Abundance of cells is detailed in the figures provided in the manuscript. Purity was determined using validated antibodies
Gating strategy	Two different panels were used to analyse the immune cell population. In all cases, cells suspensions were first pre-incubated with Fc receptor blocking solution (BioLegend) to reduce non-specific binding, and subsequently stained with Zombie aqua dye (BioLegend).<br>For the first panel, the following antibodies were used: gdTCR- Alexa421 – clone B1, CD45-PerCP Cy5 – clone 2D1, CD3-FITC – clone 17A2, CD4-PE – clone GK1.5, B220 PECy7 – clone RA3-6B2, CD8 APC Alexa647 – clone SK1 and NK1.1 APC-Cy7 – clone PK136. Cells without staining were always used as reference to distinguish between positive and negative stained cells. After excluding debris and doblets, singlets were gated using the CD45 Antibody. Cells that were CD45 and CD3 positive were counted at total T cells and were then gated using the CD4 and CD8 antibodies, to differentiate between CD4 (CD45 +ve, CD3 +ve, CD4 +ve but CD8 negative) and CD8 T cells (CD45 +ve, CD3 +ve, CD8 +ve but CD4 negative). Cells that were negative CD8 and CD4 cells, but positive for CD45, CD3 and TCR were classed as gdT cells.<br>For the second panel, the following antibodies were used: CD45-PerCP Cy5 – clone 2D1, Lyc6G Alexa 421 – clone 1A8, CD11c FITC – clon N418, CD11b PE – clon M1/70, F480 PE CY7 – clone BM8 and Siglec F-Alexa700 – clone E50-2440. Cells without staining were always used as reference to distinguish between positive and negative stained cells. After excluding debris and doblets, singlets were gated using the CD45 Antibody. CD45 positive cells were then gated using both LyC6G and CD11b. Cells that were positive for both LyC6G and CD11b were counted as neutrophils. Cells that were LyC6G negative but CD11b positive were then gate using the Siglec F antibody, and double positive cells (CD11b and Siglec F positive) were counted as eosinophils. Finally, we performed an additional gating strategy, starting this time with cells that were CD45 positive. The positive population was gated using the Siglec F and the CD11c. Cells that were positive for both markers were in turn gated for F4/80. Cells that were positive for the four markers (CD45, Siglec F, CD11c and F4/80) were classed as macrophages.<br>Representative gating plots are shown in Supplementary Figure 7

☒ Tick this box to confirm that a figure exemplifying the gating strategy is provided in the Supplementary Information.
